# Prognostic implication of neuropilin-1 upregulation in human nasopharyngeal carcinoma

**DOI:** 10.1186/1746-1596-8-155

**Published:** 2013-09-20

**Authors:** Yu Xu, Peizhong Li, Xin Zhang, Junying Wang, Dongsheng Gu, Yao Wang

**Affiliations:** 1 Department of Otorhinolaryngology, Huai’an First People’s Hospital, Nanjing Medical University, 6 Beijing Road West, Huai’an, Jiangsu 223300, P. R. China; 2 Pharmaceutical Preparation Section, Huai’an First People’s Hospital, Nanjing Medical University, 6 Beijing Road West, Huai’an, Jiangsu 223300, P. R. China

**Keywords:** Nasopharyngeal carcinoma, Neuropilin-1, Expression, Immunohistochemistry, Prognosis

## Abstract

**Objective:**

As a receptor for both vascular endothelial growth factors and semaphorin, neuropilin-1 (NRP-1) is reported to be up-regulated in cells of several cancers. However, its roles in human nasopharyngeal carcinoma (NPC) are still unclear. Therefore, the goal of this study was to investigate the expression pattern of NRP-1 in NPC tissues, to clarify the clinical significance of NRP-1 expression in NPC as well as the potential prognostic implication of NRP-1 expression.

**Methods:**

Immunohistochemistry was performed to detect the expression of NRP-1 in tumor tissue samples from 266 NPC patients. The association of NRP-1 protein expression with the clinicopathological characteristics and the prognosis of NPC were subsequently assessed.

**Results:**

Immunohistochemical analysis showed that 176 of 266 (66.17%) paraffin-embedded archival NPC biopsies showed high expression of NRP-1, but no non-cancerous nasopharyngeal specimens showed positive expression of NRP-1. In addition, high NRP-1 expression was significantly associated with advanced clinical stage (P = 0.02), positive recurrence (P = 0.001) and metastasis status (P = 0.001) of NPC. Moreover, the NPC patients with higher NRP-1 expression had shorter overall survival, whereas patients with lower NRP-1 expression had better survival (P < 0.001). Furthermore, the multivariate analysis indicated that the overexpression of NRP-1 protein was an independent prognostic factor for overall survival (P = 0.001) in NPC patients.

**Conclusion:**

These findings suggest for the first time that NRP-1 upregulation may be a novel biomarker for the prediction of advanced tumor progression and unfavorable prognosis in NPC patients who may benefit from alternative treatment strategy and targeted treatment.

**Virtual slides:**

The virtual slides for this article can be found here: http://www.diagnosticpathology.diagnomx.eu/vs/1507827881105018.

## Introduction

Nasopharyngeal carcinoma (NPC), as an Epstein-Barr virus (EBV)-related cancer, is one of the most common malignant diseases with a high prevalence in Southern China, Southeast Asia and North Africa [1]. NPC accounts for 80,000 new cases and 50,000 deaths annually in the world [2]. Different from other head and neck cancers, NPC has a high metastatic potential. The majority (75 ~ 90%) of newly diagnosed NPC patients have loco-regionally advanced disease, commonly with cervical nodal metastases [3]. Although radiotherapy alone or combined with chemotherapy is relatively successful for curing the cancer, a substantial proportion of patients do not benefit from these conventional treatments but show loco-regional recurrence, and regional lymph node and distant metastasis are the two major reasons resulted in failure of treatment in clinical practice, which suggest that NPC is no longer a problematic disease from a loco-regional control standpoint and research priorities should lie in the development of innovative strategies in order to prevent distant relapse, prolonging remission in those with metastatic disease and minimizing treatment toxicities [4]. The etiologic factors associated with NPC include genetic susceptibility, EBV infection, and other environmental factors [5]. In clinical setting, the differential diagnosis of NPC based on clinical findings would include Nasal Polyps, Non-Hodgkin Lymphoma, and Rhabdomyosarcoma. Especially, extranodal NK/T-cell lymphoma, nasal type (ENKTL), a distinct clinical pathological entity of non-Hodgkin’s lymphoma, is closely associated with EBV infection [6], which is similar to NPC. Since the exact molecular mechanism of the development and progression of NPC remains poorly understood, it is of great significance to identify potential early diagnostic markers as well as novel therapeutic targets.

Neuropilins (NRPs) are transmembrane glycoprotein receptors which are capable of binding two disparate ligands, class 3 semaphorins (Sema) and vascular endothelial growth factors (VEGF) [7]. NRPs have been demonstrated to play an important role in various biological processes, including axonal guidance, angiogenesis, tumorigenesis, and the immunologic response [8]. To data, two NRP members, NRP-1 and NRP-2, have been identified. NRP-1 is a 130- to 140-kDa single spanning transmembrane glycoprotein originally identified as a receptor for class III semaphorins (SEMA3s) mediating neuronal guidance and axonal growth [9]. Then, it was subsequently found to bind to VEGF which is a critical pro-angiogenic factor that induces proliferation and migration of endothelial cells to tumor vasculature. NRP-1 is reported to be up-regulated in cells of several cancers such as glioma [10], prostate carcinoma [11], breast cancer [12], gastric cancer [13], pancreatic carcinoma [14], colon cancer [15] and acute myeloid leukemia [16]. Recent studies have indicated that the overexpression of NRP-1 may enhance tumor angiogenesis and tumor growth in vivo. In contrast, the inhibition of NRP-1 may suppress cell survival and migration [17]. However, it roles in human nasopharyngeal carcinoma (NPC) are still unclear. Therefore, the goal of this study was to investigate the expression pattern of NRP-1 in NPC tissues, to clarify the clinical significance of NRP-1 expression in NPC as well as the potential prognostic implication of NRP-1 expression.

## Materials and methods

### Patients and tissue samples

The study was approved by the Research Ethics Committee of Ministry of Public Health of China. Informed consent was obtained from all of the patients.

Two hundred and sixty-six archival formalin-fixed, paraffin-embedded NPC specimens and 100 non-cancerous nasopharyngeal specimens were obtained from Huai’an First People’s Hospital, Nanjing Medical University during 1996 ~ 2006. In the 266 NPC patients, there were 166 male and 100 female with age ranging from 16 to 80 years (median, 48 years). None of the patients recruited in this study had chemotherapy or radiotherapy before the surgery. Clinical information was obtained by reviewing the medical records on radiographic images, by telephone or written correspondence, and by review of death certificate. All specimens had confirmed pathological diagnosis and were staged according to the 1992 NPC staging system of China. Clinical characteristics of these patients are summarized in Table 1.

**Table 1:** Association of NRP-1 expression with clinicopathologic parameters of nasopharyngeal carcinoma patients

**Parameters**	**N**	**NRP-1 expression (n, %)**		**P**
		**High**	**Low**	
**Age**				
< 48	100	63 (63.00)	37 (37.00)	NS
≥ 48	166	113 (68.07)	53 (31.93)	
**Gender**				
Male	166	106 (63.86)	60 (36.14)	NS
Female	100	70 (70.00)	30 (30.00)	
**Clinical stage**				
I ~ II	68	30 (44.12)	38 (55.88)	0.02
III ~ IV	198	146 (73.74)	52 (27.26)	
**T-stage**				
T1 ~ T2	90	55 (61.11)	35 (38.89)	NS
T3 ~ T4	176	121 (68.75)	55 (31.25)	
**N-stage**				
N0	78	50 (64.10)	28 (35.90)	NS
N1 ~ N3	188	126 (67.02)	62 (32.98)	
**WHO histological type**				
II	76	50 (65.79)	26 (34.21)	NS
III	190	136 (71.58)	54 (28.42)	
**Recurrence**				
No	196	116 (59.18)	80 (40.92)	0.001
Yes	70	60 (85.71)	10 (14.29)	
**Metastasis**				
No	211	128 (60.66)	63 (39.34)	0.001
Yes	55	48 (87.27)	7 (12.73)	

‘NS’ refers to the difference without statistical significance.

For the analysis of survival and follow-up, the date of surgery was used to represent the beginning of the follow-up period. All the patients who died from diseases other than NPC or from unexpected events were excluded from the case collection. Follow-ups were terminated until March 8, 2012. The median follow-up period was 46 months (range, 3–126 months). Treatment modalities after relapse were given according to a uniform guideline as described.

### Immunohistochemistry analysis

Expression pattern of NRP-1 protein in 266 NPC specimens and 100 non-cancerous nasopharyngeal specimens was detected by immunohistochemical staining. In brief, all tissue specimens were retrieved and cut into 4-μm sections and mounted on pre-coated slides. After deparaffinizing in xylene and washing in a graded series of ethanol, Sections were submerged into EDTA antigenic retrieval buffer and microwaved for antigenic retrieval. The slides were incubated with the primary antibody against NRP-1 (rabbit polyclonal anti-human NRP-1, 1:100 dilutions, Zymed No. 34–7300, San Francisco, CA, USA). All incubations with primary antibody were carried out overnight at 4°C. After washing in TBS (Sigma, Munich, Germany), the tissue sections were treated with biotinylated anti-rabbit secondary antibody (Zymed, San Francisco, CA), followed by further incubation with streptavidinhorseradish peroxidase complex (Zymed). The tissue sections were immersed in 3-amino-9-ethyl carbazole and counterstained with 10% Mayer’s hematoxylin, dehydrated, and mounted in Crystal Mount. In each immunohistochemistry run, non-cancerous nasopharyngeal specimens were used as control tissues and omission of the primary antibody served as negative control. All tissue samples were stained at one time.

Following a hematoxylin counterstaining, immunostaining was scored by two independent observers, who were blinded to the clinicopathological parameters and clinical outcomes of the patients. The scores of the two observers were compared and any discrepant scores were trained through re-examining the stainings by both pathologists to achieve a consensus score. The number of positive-staining cells showing immunoreactivity on the cell membrane and/or cytoplasm (for NRP-1) in ten representative microscopic fields was counted and the percentage of positive cells was calculated. The frequency of NRP-1 immunoreactivity in tissue sections was evaluated as negative (0) when no positive cells were observed within the tumor, weak (1) when < 30% of the tumor cells were positive, moderate (2) when 30% to 60% of the tumor cells were positive, and strong (3) when >60% of tumor cells were positive. The intensity of staining was evaluated as 0, 1, 2, and 3 for no staining, weak staining, medium staining, and strong staining, respectively. Immunohistochemical scores were determined as the sum of the frequency and intensity score for tumor cells. Cutoff values for immunohistochemical scores of NRP-1 protein were chosen on the basis of a measure of heterogeneity with the log-rank test statistic with respect to overall survival. An optimal cutoff value was identified. An immunohistochemical score of ≥ 4 was used to classify tumors with high expression, and an immunohistochemical score < 4 was used to classify tumors with low expression of NRP-1 antigen.

### Statistical analysis

The software of SPSS version 16.0 for Windows (SPSS Inc, IL, USA) and SAS 9.1 (SAS Institute, Cary, NC) was used for statistical analysis. Continuous variables were expressed as $\bar{X}\pm s$. Associations between the expression of NRP-1 and clinicopathological parameters were assessed using a Chi-Square test. Survival curves were plotted by Kaplan-Meier analysis and compared by the log-rank test. Cox regression analysis was performed to assess the significance of various variables for survival. Differences were considered statistically significant when *p* was less than 0.05.

## Results

### Upregulation of NRP-1 in human NPCs

**Figure 1:**
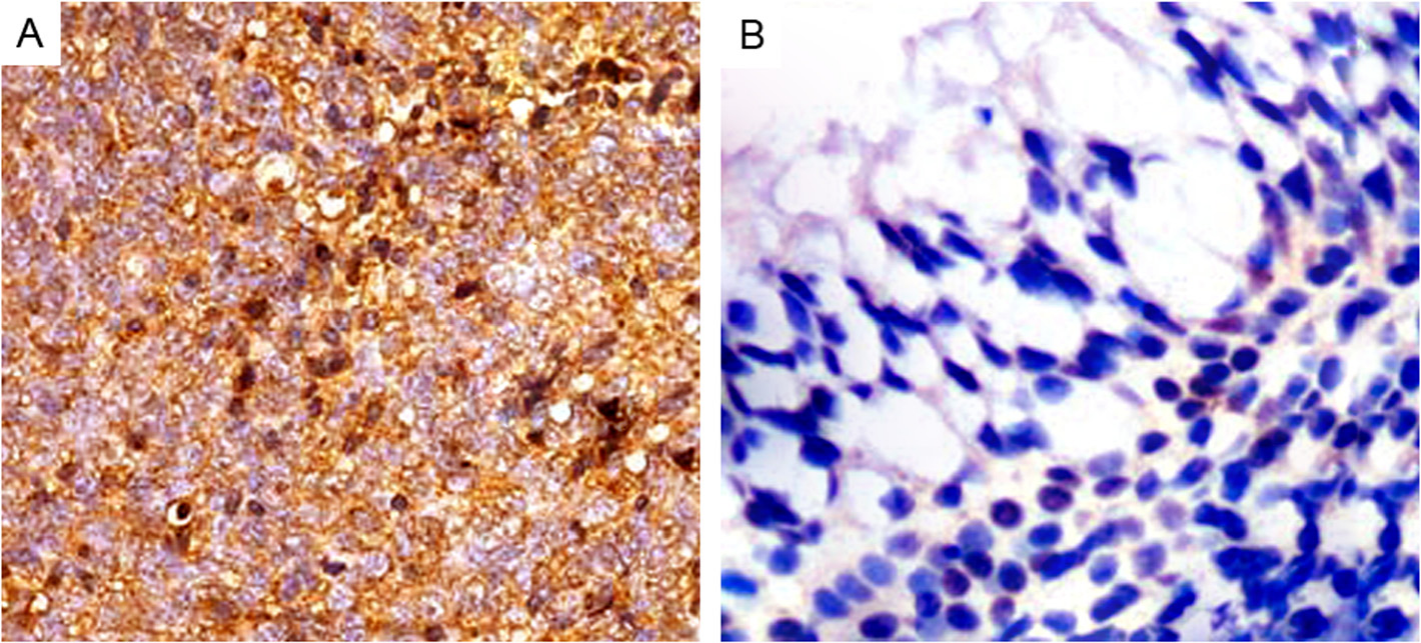
**Immunohistochemical staining of NRP-1 protein in tumor cells of nasopharyngeal carcinoma (NPC, A) and non-cancerous nasopharyngeal tissues (B; Original magnification × 400).** Intense staining of NRP-1 is seen in the cell membrane and/or cytoplasm of tumors cells and is intensive in NPC tissues **(A)**. In contrast, negative immunostainings of NRP-1 **(B)** was observed in non-cancerous nasopharyngeal tissues.

Expression and subcellular localization of NRP-1 protein was detected by immunohistochemistry in 266 NPC specimens and 100 non-cancerous nasopharyngeal specimens. The coefficient of variation of interobserver reproducibility was 9.62% for NRP-1 expression scoring, with no significant differences between the two observers (paired *t*-test, P = 0.82). As shown in Figure 1, the cell membrane and/or cytoplasm of most tumor cells in the NPC sections stained intensely with NRP-1 antibody (Figure 1A), while negative immunostainings were observed in all non-cancerous nasopharyngeal tissues (Figure 1B). High NRP-1 expression was observed in tumor cells of 66.17% (176/266) NPCs.

### Association of NRP-1 protein expression with the clinicopathological characteristics of human NPCs

Table 1 summarized the association of NRP-1 protein expression in 266 NPCs detected by immunohistochemical staining. High NRP-1 expression is closely associated with advanced clinical stage of NPC patients (P = 0.02). In addition, there was a significant difference of NRP-1 expression in patients categorized according to recurrence status (P = 0.001). The expression levels of NRP-1 protein in NPC patients with positive recurrence were significantly higher than those without recurrence. Moreover, positive metastasis also correlated with higher NRP-1 expression (P = 0.001). No significant association between NRP-1 expression and age, gender, T-stage, N-stage or WHO histological type was observed (Table 1).

### Association of NRP-1 protein expression with the prognosis of human NPCs

**Figure 2:**
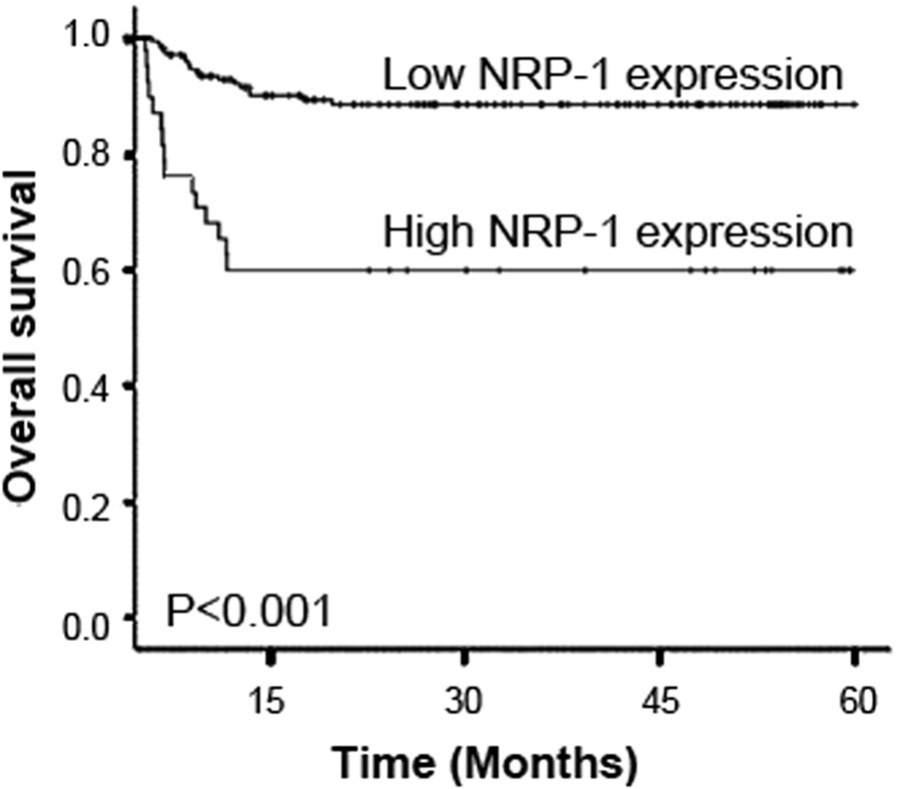
**Kaplan-Meier survival plots of NRP-1 protein expression.** Kaplan-Meier survival analysis revealed that the NPC patients overexpressing NRP-1 protein exhibited markedly poorer overall survival (P < 0.001).

The association of NRP-1 protein expression with the prognosis of human NPCs was also evaluated. The 5-year overall survival rate of the cohort of 266 NPC patients was 66.17% (196/266). Kaplan-Meier analysis and the log-rank test were used to calculate the effect of classic clinicopathologic characteristics (including age, gender, clinical stage, T-stage, N-stage, WHO histologic type, recurrence and metastasis) and NRP-1 expression on survival. The log-rank test showed that the low NRP-1 expression group had better survival, whereas the high NRP-1 expression group had shorter survival (P < 0.001, Figure 2). In addition, clinical stage, recurrence and metastasis were also significantly correlated with survival in Kaplan-Meier analysis and log-rank test (for clinical stage, P = 0.002; for recurrence and metastasis, P = 0.001, Table 2). Moreover,we did multivariate survival analysis, which included NRP-1 expression level, clinical stage, recurrence and metastasis, to determine if NRP-1 expression level is an independent prognostic factor of outcomes. In this analysis, clinical stage (HR = 6.193, 95% CI: 1.011-13.392, P = 0.006), recurrence (HR = 6.928, 95% CI: 0.922-13.556, P = 0.005), metastasis (HR = 6.893, 95% CI: 1.023-13.528, P = 0.005), and NRP-1 expression (HR = 8.631, 95% CI: 1.392-18.288, P = 0.001) were recognized as independent prognostic factors for NPC patients (Table 3).

**Table 2:** Univariate analysis of different prognostic varibles in 266 nasopharyngeal carcinoma patients

**Variables**	**Subset**	**Hazard ratio (HR)**	**95% confidence interval [CI]**	**P**
**Patient gender**	Male vs. Female	1.987	0.608 ~ 4.092	NS
**Patient age**	< 48 vs. ≥ 48	1.566	0.465 ~ 3.853	NS
**Clinical stage**	I ~ II vs. III ~ IV	7.382	1.031-16.613	0.002
**T-stage**	T1 ~ T2 vs. T3 ~ T4	2.380	0.738 ~ 5.046	NS
**N-stage**	N0 vs N1 ~ N3	1.778	0.732 ~ 4.028	NS
**WHO histological type**	II vs. III	1.458	0.689 ~ 3.076	NS
**Recurrence**	No vs. Yes	8.892	1.021-18.656	0.001
**Metastasis**	No vs. Yes	8.788	1.042-19.052	0.001
**NRP-1**	Low vs. High	16.926	1.833-38.132	< 0.001

‘NS’ refers to the difference without statistical significance.

**Table 3:** Multivariate cox regression analysis of different prognostic varibles in 266 nasopharyngeal carcinoma patients

**Variables**	**Subset**	**Hazard ratio (HR)**	**95% confidence interval [CI]**	**P**
**Clinical stage**	I ~ II vs. III ~ IV	6.193	1.011-13.392	0.006
**Recurrence**	No vs. Yes	6.928	0.922-13.556	0.005
**Metastasis**	No vs. Yes	6.893	1.023-13.528	0.005
**NRP-1**	Low vs. High	8.631	1.392-18.288	0.001

## Discussion

NPC is a malignancy with significant geographic and racial distributions worldwide. Carcinogenesis of NPC is a multistep process with morphological progression involving multiple genetic and epigenetic events. Therefore, it is of great significance to identify the molecular and biological changes that occur during carcinogenesis and progression, in order to facilitate the investigation of the pathology of the disease and generate new prognostic markers to more accurately screen patients who are at the great risk of relapse after their primary treatment. In the present study, we analyzed the expression of NRP-1 with the clinicopathologic parameters in NPC patients. We observed the overexpression of NRP-1 in NPC tissues to be associated with advanced clinical stage, positive recurrence and metastasis status, and poor prognosis, suggesting that the overexpression of NRP-1 may be a novel independent marker for progression and prognosis in NPC patients. To the best of our knowledge, this is the first research on the role of NRP-1 expression in a large cohort of NPC patients.

NRP-1 is a single-spanning transmembrane glycoprotein with a large extracellular domain and a short cytoplasmic tail [18]. It has been known to have an important role in angiogenesis as a VEGFR and is involved in neuronal guidance during embryogenesis [19]. According to the experiments in vivo, the overexpression of NRP-1 in a transgenic mouse model may increase capillary and blood vessel formation leading to hemorrhage, whereas functional inactivation of NRP-1 in mice may result in embryonic lethality with multiple vascular abnormalities, including of avascular regions, heterogeneous blood vessel size and abnormally formed dorsal aorta [20]. These findings suggest that NRP-1 may be a key regulator of developmental angiogenesis. Pathologically, the upregulation of NRP-1 has been found in a variety of tumor cells and has been demonstrated to be involved in tumorigenesis and tumor progression. For example, Meyerson et al. [21] reported that NRP-1 was overexpressed in precursor B-cell acute lymphoblastic leukemia samples compared with normal B-cell progenitors, allowing for its potential use as a marker for the detection of minimal residual disease; Li et al. [22] demonstrated that NRP-1 was overexpressed in glioblastoma and may be an attractive candidate as a therapeutic target for this disease; Xu et al. [23] indicated that NRP-1 may be able to promote the growth of hepatocellular carcinoma in vitro and in vivo, and therefore may be considered as a novel therapeutic target for this carcinoma; Hansel et al. [24] found the increased expression of NRP-1 in gastrointestinal adenocarcinomas and in a subset of high-grade precursor lesions, which appears to parallel invasive behavior and may therefore be used as a potential marker for cancer aggressiveness; Baba et al. [25] reported that the enhanced expression of NRP-1 may be not only associated with oncogenesis, but also with ovarian cancer malignancy, and this molecule may be a targeting candidate for the treatment of ovarian malignancies; Stephenson et al. [26] also suggested that the upregulation of NRP-1 may be involved in the induction of local invasiveness of neoplasia and angiogenesis and have direct relevance to the progression of breast cancer. In line with these previous studies, our immunohistochemistry analysis also found the overexpression of NRP-1 in NPC tissues compared with non-cancerous nasopharyngeal tissues. More importantly, the upregulation of NRP-1 was significantly associated with the advanced clinical stage, positive recurrence and metastasis status of NPC patients. It is worth noting that overexpression of NRP-1 protein was found to be an indicator of poor prognosis in NPC patients, and NRP-1 expression, clinical stage, recurrence and metastasis were independent predictors of NPC. However, in colon cancer, Kamiya et al. [27] found that the gene expression levels of NRP-1 were significantly decreased compared to those in the extra-neoplastic tissues, and the preserved NRP-1 expression provides colon cancer patients with a better prognosis. These results suggest that NRP-1 has cancer-type dependent expression patterns in various cancers and the abnormal expression of NPR-1 may play important roles in tumor progression and tumor prognosis.

In conclusion, our data show for the first time that NRP-1 upregulation may be a novel biomarker for the prediction of advanced tumor progression and unfavorable prognosis in NPC patients who may benefit from alternative treatment strategy. These findings support the hypothesis of an important role for NRP-1 in aggressive progression of NPC, so that targeting of NRP-1 could constitute a novel strategy in the treatment of advanced NPC. Future research should address this interesting issue further.

## Competing interests

The authors declare that they have no competing interests.

## Authors’ contributions

YX carried out the experimental design, participated in the data analysis and drafted the manuscript; PL participated in the experiments and data analysis; XZ and JW participated in the experiments. DG and YW participated in the data analysis. All authors read and approved the final manuscript.
